# Global Biodiversity and Phylogenetic Evaluation of Remipedia (Crustacea)

**DOI:** 10.1371/journal.pone.0019627

**Published:** 2011-05-19

**Authors:** Marco T. Neiber, Tamara R. Hartke, Torben Stemme, Alexandra Bergmann, Jes Rust, Thomas M. Iliffe, Stefan Koenemann

**Affiliations:** 1 Institute for Animal Ecology and Cell Biology, University of Veterinary Medicine Hannover, Hannover, Germany; 2 Division of Cell Biology, Institute of Physiology, University of Veterinary Medicine Hannover, Hannover, Germany; 3 Department of Palaeontology, Steinmann Institute, University of Bonn, Bonn, Germany; 4 Department of Marine Biology, Texas A&M University at Galveston, Galveston, Texas, United States of America; 5 Section of Biology, Science and Technology, University of Siegen, Siegen, Germany; Paleontological Institute of Russian Academy of Science, United States of America

## Abstract

Remipedia is one of the most recently discovered classes of crustaceans, first described in 1981 from anchialine caves in the Bahamas Archipelago. The class is divided into the order Enantiopoda, represented by two fossil species, and Nectiopoda, which contains all known extant remipedes. Since their discovery, the number of nectiopodan species has increased to 24, half of which were described during the last decade. Nectiopoda exhibit a disjunct global distribution pattern, with the highest abundance and diversity in the Caribbean region, and isolated species in the Canary Islands and in Western Australia. Our review of Remipedia provides an overview of their ecological characteristics, including a detailed list of all anchialine marine caves, from which species have been recorded. We discuss alternative hypotheses of the phylogenetic position of Remipedia within Arthropoda, and present first results of an ongoing molecular-phylogenetic analysis that do not support the monophyly of several nectiopodan taxa. We believe that a taxonomic revision of Remipedia is absolutely essential, and that a comprehensive revision should include a reappraisal of the fossil record.

## Introduction

**Figure 1:**
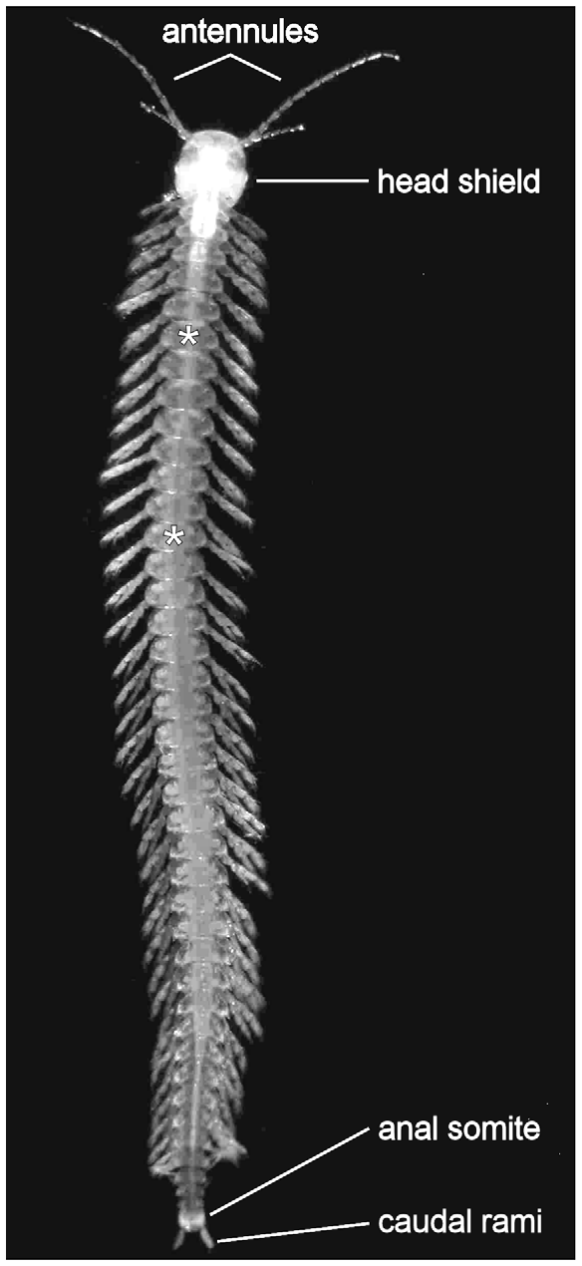
Habitus of a remipede. Photograph of a living specimen of *Speleonectes tanumekes* from the Exuma Cays, Bahamas; asterisks indicate the location of female and male gonopores on trunk somites seven and 14, respectively (Photograph courtesy of J. van der Ham).

Remipedia Yager, 1981 is one of the most recently discovered classes of crustaceans, first collected in 1979 from an anchialine cave system (see below) on Grand Bahama Island [1]. All extant remipedes are (probably simultaneous) hermaphrodites, with female genital pores on the protopods of the seventh trunk limbs, and male gonopores opening on the fourteenth trunk limbs. Similar to many other hypogean animals, remipedes are pale and eyeless. Their body is made up of two main regions, a cephalon and a long homonomous trunk lacking tagmosis (Figure 1). Remipedes do not have a carapace. The head has six appendage-bearing somites, including a pair of maxillipeds, and is covered by a chitinous, ovoid to trapezoidal, dorsal shield. The long biramous antennules (first antennae) serve as cephalic sensory appendages. Short, paired filamentous processes, found on the ventroanterior margin of the head shield between the antennules, are presumably also sensory structures [2]. The small biramous antennae (second antennae) do not have any apparent sensory function. Posterior to the asymmetrical, palp-less mandibles, the uniramous maxillules, maxillae, and maxillipeds are developed as prehensile, raptorial mouthparts.

All trunk segments are equipped with a pair of paddle-shaped biramous swimming appendages. The posterior-most trunk somite has a terminal anus, and bears a pair of simple caudal rami. The trunk segments and their limbs become smaller toward the posterior body region. Limb buds on these segments suggest that adults continue to grow and add segments their entire lives [3]. The greatest number of 42 trunk segments was counted in an as yet undescribed species from the Yucatán Peninsula [3]. Adult body length is approximately 9 mm in small species and up to 45 mm in larger species.

All known remipedes inhabit submerged marine (anchialine) caves, accessible only to highly-trained cave divers. While our knowledge of remipedes has increased greatly, particularly over the last ten years, there are still large gaps in our understanding of their ecology, ethology and evolutionary history. For example, nothing is yet known about their mating habits. It has been speculated that fertilization must be external, as the constant motion of the trunk limbs, even during a resting state, would interfere with copulation [4]. Larval forms were discovered as recently as 2006 [4], [5], however the sequence of pre- and postembryonic development still has several gaps.

Remipedes are often described as “enigmatic”, reflecting, to some extent, the difficulty of collecting and observing them. However, it is their unique body plan, composed of a head with six fused, appendage-bearing somites and an undivided, homonomously segmented trunk that makes remipedes stand out among the disparity of crustacean shapes and forms. All major extant groups of Crustacea Brünnich, 1772 [6] feature a division of their trunks (the body region posterior to the head) into at least two functionally and morphologically different tagmata, for example, thorax and pleon, or thorax and abdomen [7]. Accordingly, an undivided trunk has been regarded as a basal or “primitive” character in crustaceans [8].

Early phylogenetic analyses based on morphological data sets reflect these assumptions about “primitive” and “derived” morphological traits; remipedes were either chosen a priori as an outgroup [8] or emerged at a basal position within clades composed of extant crustaceans [9], [10]. However, the advance of molecular sequence analysis and comparison of neuroanatomical data contradicted the presumed basal position of Remipedia (see Higher-level classification and phylogenetic relationships). Although we have not reached consensus yet, an impressive number of independent studies suggest that remipedes represent a highly derived group of pancrustaceans phylogenetically related to malacostracans and/or hexapods (see below). Our first results of an ongoing molecular-phylogenetic analysis suggest a sister-group relationship between Remipedia and Cephalocarida Sanders, 1955 [11], a clade that has also been recovered in two recent studies [12], [13]. However, the analysis based on CO1 sequence data does not support monophyly of the families Godzilliidae Schram et al., 1986 [14] and Speleonectidae Yager, 1981 [1], and the genera *Speleonectes* Yager, 1981 [1] and *Lasionectes* Yager and Schram, 1986 [15].

### Ecology

Almost all species of Remipedia have been found exclusively in anchialine cave systems. Anchialine caves are located in coastal regions; on the landside, they are affected by both terrestrial freshwater input and tidal exchange with ocean waters via subsurface channels and cracks. Known as Blue Holes on the Bahamas, and Cenotes on the Yucatán Peninsula, anchialine limestone caves typically connect to freshwater or brackish ponds at the surface. Nearly all remipedes live in the deepest parts of the caves in the seawater zone below the halocline. The only known species that inhabits a fully marine, sub-seafloor cave, *Speleonectes kakuki* Daenekas et al., 2009 [16], has been described from Andros, Bahamas.

This marine cave habitat is characterized by low nutrient availability and small population sizes of the organisms living there. Remipedes are typically found in cave sections with low oxygen (<1 ppm), salinity generally around 35 ppt, but in some cases as low as 18 ppt, and temperatures ranging from 22 to 26°C [17]. One exception, *Speleonectes epilimnius* Yager and Carpenter, 1999 was collected from the highly oxygenated (3 to 5 mg/l) surface water of an anchialine cave on San Salvador, southeastern Bahamas [18], [19].

Remipedes have been observed consuming shrimp of the genus *Typhlatya* Creaser, 1936 [20], and are thought to be scavengers and top predators in the ecosystems in which they are found. In Crustacea Cenote on the Yucatán Peninsula, remipedes have been frequently observed swimming just above the floor of the cave (pers. obs., TMI), where they are thought to feed. Other macroorganisms that have been reported to co-occur with remipedes include polychaete worms, ostracodes, amphipods, isopods, mysids, thermosbaenaceans, copepods, shrimp, and cave fish.

Microorganisms are also important members of anchialine cave ecosystems, and their interactions with remipedes are not yet fully understood. The microbial community in anchialine cave systems, most conspicuously represented by wispy to dense bacterial clouds floating in a hydrogen sulphide layer at the halocline and thick bacterial mats on the rock walls and floors of some caves, are currently being studied (pers. comm. M. J. Pakes, B. Gonzalez). Moreover, epibionts have been observed on some remipedes, including suctorians, rod-shaped bacteria, and unidentified protists. Gregarines are present in the gut, and rod-shaped bacteria have been reported throughout the tissues [21], [22].

Remipedes and their habitat are starting to be protected. The Australian Cape Range remipede, *Lasionectes exleyi* Yager and Humphreys, 1996 [23], is the object of conservation measures, and serves as an indicator species for the health of Bundera Sinkhole [24]. Remipedes are also protected within the Lucayan National Park in the Bahamas, and there are efforts to protect remipede habitat on Abaco and Andros Islands in the Bahamas, and on the Yucatán Peninsula. Cave divers are reducing their use of open circuit diving systems, which release exhaust gasses that increase dissolved oxygen in the water and change the microbial community in anchialine cave ecosystems. The use of closed circuit rebreathers, which recycle exhaled gas and do not release bubbles, are important to the health of remipede habitats [25].

### Higher-level classification and phylogenetic relationships

While we have probably reached a general consensus that Remipedia represent a derived rather than a primitive group, their phylogenetic position within the arthropods is far from clear. Competing hypotheses have placed remipedes as a sister group to all other crustaceans [9], [26], cephalocarids [12], [13], [27]–[29], cirripedes [30], [31], malacostracans [2], [4], [32], collembolans [33], and diplurans [31] (see also review by [34]). This listing is not exhaustive and a critical evaluation of individual results should consider the choice of molecular markers and methodical approaches.

**Figure 2:**
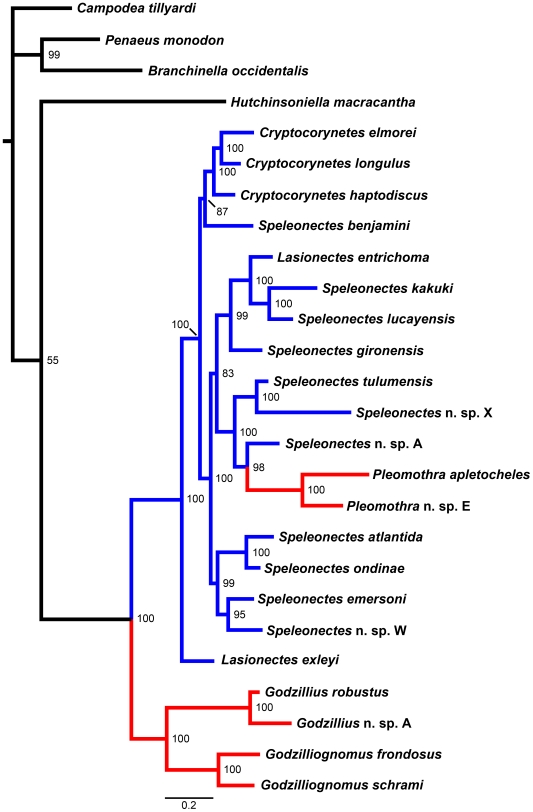
50% majority-rule consensus tree of Remipedia and outgroup taxa based on a Bayesian analysis of CO1 sequence data. Bayesian posterior probability values of clades are noted at the nodes of the tree. Remipede lineages currently assigned to the family Speleonectidae and Godzilliidae are indicated in blue and red, respectively. Outgroup lineages are indicated in black.

Interestingly, numerous independent investigations, using a rather diverse selection of data types, have found a sister group relationship between remipedes (in some cases together with cephalocarids) and hexapods. For example, remipede-hexapod affinities have been suggested based on morphological data [35], brain architecture [2], [32], hemocyanin sequences [36], and various combinations of nuclear and mitochondrial genes [12], [13], [27], [31], [37]. The results of our Bayesian analysis of CO1 sequences from 22 remipedes and four hexapod and crustacean species show a weakly-supported sister-group relationship between Remipedia and Cephalocarida, while the relationship between (Remipedia+Cephalocarida) and the remaining outgroup taxa, Hexapoda Blainville, 1816 [38] and (Malacostraca Latreille, 1802 [39]+Branchiopoda Latreille, 1817 [40]), remains unresolved (Figure 2; Material and methods section). However, we consider this result as preliminary, since a phylogenetic evaluation of higher-level outgroup taxa should include additional, more conserved markers.

**Figure 3:**
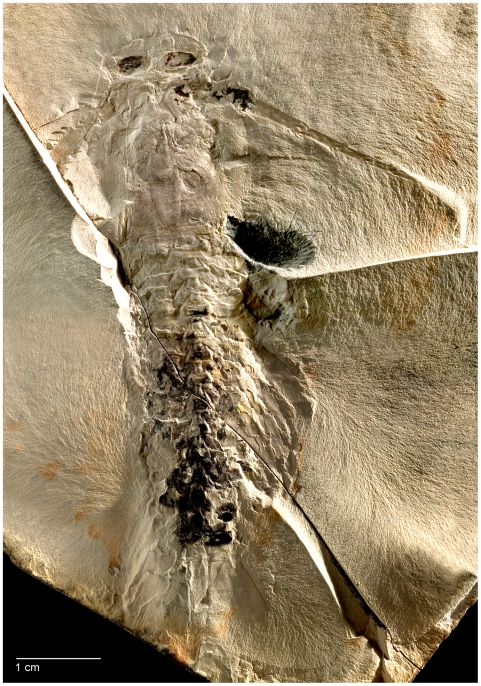
Holotype of *Tesnusocaris goldichi* (Remipedia, Enantiopoda). The holotype (catalogue number USNM 124173a) has a length of approximately 77 cm; it was collected by S. S. Goldich (1939) in the Tesnus Formation (Pennsylvanian), West of Rough Creek, Dove Mountain Quadrangle, Brewster County, Texas.

**Figure 4:**
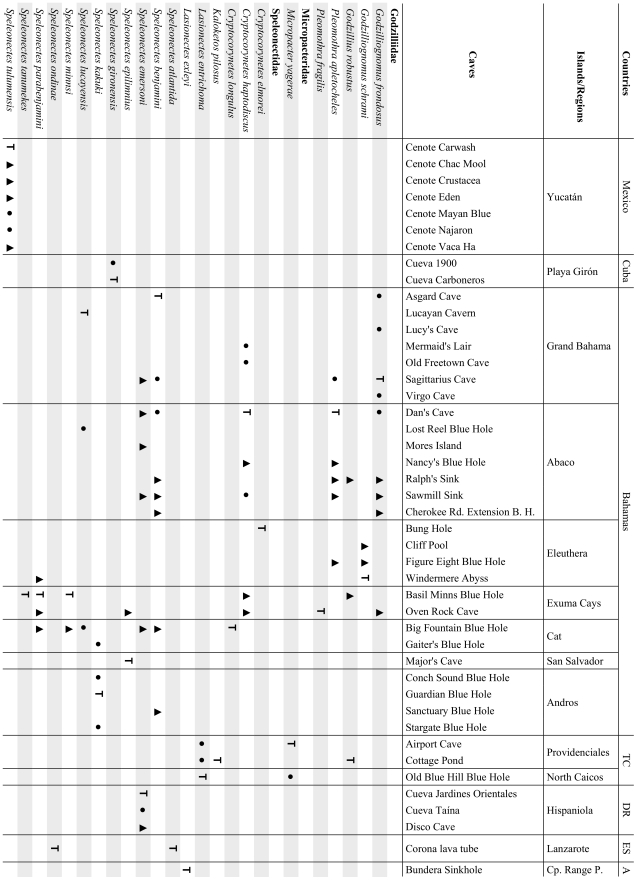
Distributional records of nectiopodan remipedes. Included in the list are all caves with confirmed occurrence of Remipedia. Type localities for species are indicated by (T) and confirmed additional records by a black dots. Records of Remipedia, which are morphologically similar to the respective species, but either need to be confirmed or may represent cryptic species, are referred to by triangles. Abbreviations: A: Australia; DR: Dominican Republic; ES: Spain; TC: Turks and Caicos; B. H.: Blue Hole; Rd.: Road; Cp.: Cape; P.: Peninsula.

The class Remipedia embraces two orders, the extinct Enantiopoda Birshtein, 1960 [41] and Nectiopoda Schram, 1986 [9]. Enantiopoda includes the fossil species *Tesnusocaris goldichi* Brooks, 1955 (Figure 3) and *Cryptocaris hootchi* Schram, 1974, both placed in the family Tesnusocaridae Brooks, 1955 (see [42]–[44] and Fossil Record below). Nectiopoda contains all known extant remipedes and is divided into the three families Speleonectidae, Godzilliidae, and Micropacteridae Koenemann et al., 2007 [45], with a total of eight genera and 24 described species (Figure 4). The taxonomic classification of Remipedia is chiefly based on morphological descriptions and diagnoses of taxa from the 1980s, when only a small number of species was known. Since 2002, the number of species has doubled, and with the addition of new taxa, morphological definitions of families and some genera are subject to a great deal of uncertainty.

In our Bayesian analysis of CO1 sequences from 22 remipede species, almost all clades within Remipedia are highly to fully supported (Figure 2). Two of the three currently recognized families, Godzilliidae and Speleonectidae, emerge as paraphyletic assemblages. Unfortunately, there are to date no CO1 data available from the monotypic family Micropacteridae. The godzilliid genus *Pleomothra* Yager, 1989 [46] is deeply nested and fully supported within a large clade composed of speleonectids. Within this large clade, the comparatively species-rich genus *Speleonectes* and the small genus *Lasionectes* are recovered as paraphyletic groups. Interestingly, the disjunct Australian species *Lasionectes exleyi* emerges as a basal sister-group to the large clade composed of all remaining speleonectids and *Pleomothra*.

Our analysis of CO1 sequences suggests that the current taxonomic structure of Remipedia does not accurately reflect the phylogeny of the class. Apparently, current ideas about morphological apomorphies such as the modification of the prehensile cephalic limbs need to be reconsidered. Preliminary analyses of additional sequence data (not shown), including the protein-encoding nuclear gene H3 and the ribosomal markers 18S and 16S, are in general agreement with the results obtained from CO1. At present, we are preparing a taxonomic revision of Remipedia based on phylogenetic analyses of these markers and a comprehensive re-evaluation of morphological characters; our revision will also include a reappraisal of the fossil taxa assigned to the class.

### Fossil Record

The fossil record of Remipedia is extremely poor. All known enantiopodan specimens are classified as either *Tesnusocaris goldichi* or *Cryptocaris hootchi*. *Tesnusocaris goldichi* was discovered in 1939 by S. S. Goldich in the Tesnus Formation of the Marathon region of Western Texas [42]. The Tesnus Formation is a mountain stump of the Paleozoic Appalachian orogeny, built of about 1850 m of alternating shales and sandstones marking the transition from the Mississippian to the Pennsylvanian subperiod in the Carboniferous [47]. *Tesnusocaris goldichi* was first examined by Brooks in 1955 [42]. The holotype is preserved in a calcareous claystone concretion (Figure 3). The specimen has a cephalic tagma with a dorsal, anteriorly rounded head shield that bears large elliptical compound eyes; its trunk is composed of homonomous segments that decrease in size posteriorly. Brooks described *Tesnusocaris goldichi* as having a thin unornamented, chitinous exoskeleton. He distinguished five pairs of appendages on the head, and identified 15 strongly chitinized sternites on the trunk somites, each bearing a pair of spatulate, seven-jointed appendages. In his study, Brooks also proposed a possible phylogenetic relationship of *Tesnusocaris goldichi* to the Branchiopoda, but in a footnote he also discussed a possible relationship between *Tesnusocaris goldichi* and the then newly-erected subclass Cephalocarida Sanders, 1955 [11], on the basis of an unspecialized postcephalic tagma and the presence of jointed appendages.

In 1985, an expedition to the type locality of *Tesnusocaris goldichi* by Emerson and Schram [44], [47] yielded five additional fossils, three of which provided sufficient details for a reconstruction. The authors interpreted the specimens as juveniles and placed them in the genus *Tesnusocaris*. Emerson and Schram assigned a second species, *Cryptocaris hootchi*, to the order Enantiopoda on the basis of presumed features shared with *Tesnusocaris*. These included a homonomously segmented trunk, a simple head shield, large biramous antennules with different segmentation on the two rami, long annulate caudal rami and large raptorial mouthparts [44]. To date, only four specimens of *Cryptocaris hootchi* are known. The holotype was collected in Upper Carboniferous (Middle Pennsylvanian) Francis Creek Shale deposits in Will County, Illinois [43], [44]. Because of the incomplete preservation of the available fossils, the authors excluded *Cryptocaris hootchi* (for the most part) from their reconstruction and discussion of Enantiopoda.

Emerson and Schram suggested that each trunk segment of *Tesnusocaris goldichi* bore two pairs of uniramous, paddle-shaped limbs, a feature they termed “duplopody” [44]. As a consequence, the authors proposed that the trunk limbs of *Tesnusocaris* are not secondary modifications of a primarily biramous appendage, but rather that the biramous trunk limbs of Nectiopoda may have evolved from duplopodous appendages through fusion of two uniramous limbs at their basis to form a protopod with two distal rami. In a subsequent paper, Emerson and Schram [48] extended their hypothesis and discussed the possibility that biramous limbs in Crustacea and probably all arthropods evolved from the basal fusion of duplopodous, uniramous appendages. They tested their hypothesis in the framework of a phylogenetic analysis, focusing in particular on the nature of trunk limbs. The only two taxa displaying duplopody in Emerson and Schram's tree are *Tesnusocaris goldichi* and *Branchiocaris pretiosa* (Resser, 1929) [49], [50]. In the analysis with unordered character states, this character appears as an autapomorphy. The duplopody hypothesis thus remains highly speculative because of limited evidence among arthropods.

**Figure 5:**
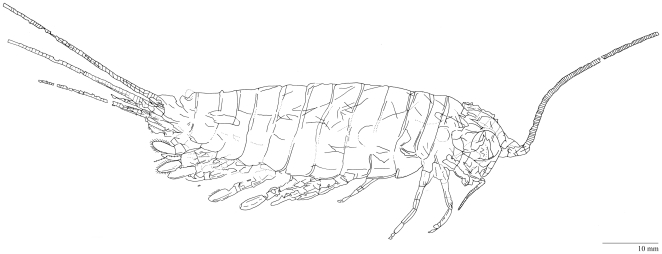
Camera lucida drawing of specimen SDNHM 28852. Collected by Emerson and Schram (1985) at the type locality of *Tesnusocaris goldichi* (see Figure 3).

*Tesnusocaris goldichi* emerged in several phylogenetic analyses of arthropods within a (pan-)crustacean clade (see, e.g., [5]). In the phylogenetic analysis of Wills [6], recent Nectiopoda together with the extinct Enantiopoda were resolved basally within Crustacea; however, the author designated Remipedia a priori as an outgroup in his analyses, and rooted his trees by them, thus biasing the results. From our initial examination of the holotype and subsequently discovered specimens, we think that many aspects of the morphological reconstruction of *Tesnusocaris* might be questionable and require further investigations. First results of an ongoing re-examination of the holotype and the additional fossils discovered by Emerson and Schram [43], [47] suggest that only one of the additional fossils (SDNHM 28852; Figure 5) represents an arthropod. The two other evaluable specimens, each with plate and counterplate, are most likely remains of polychaetes. Specimen SDNHM 28852 exhibits conspicuous morphological differences in both cephalic and trunk appendages when compared to the holotype of *Tesnusocaris goldichi* (Figure 3).

### Extant Remipedia: diversity and distribution

**Figure 6:**
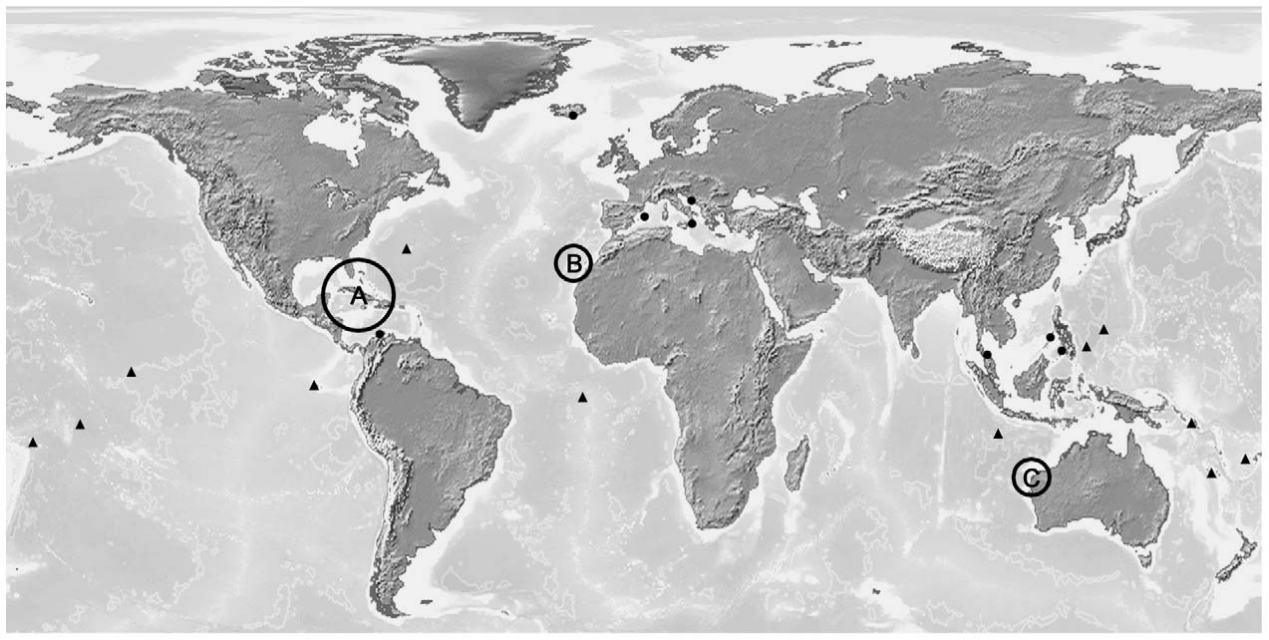
Global distribution of anchialine caves. Epicontinental anchialine cave systems are indicated by dots and anchialine waters on isolated seamount islands by triangles. Remipedia show a disjunct global distribution pattern, with all known species restricted to epicontinental anchialine caves. The majority of remipede species inhabit the larger Caribbean region (A), including the Yucatán Peninsula, Cuba, the Dominican Republic, the Turks and Caicos Islands and the Bahamas. Isolated species occur in caves on the Canarian Island of Lanzarote (B) and in Western Australia (C). Map (modified) with kind permission of Demis (www.demis.nl).

**Figure 7:**
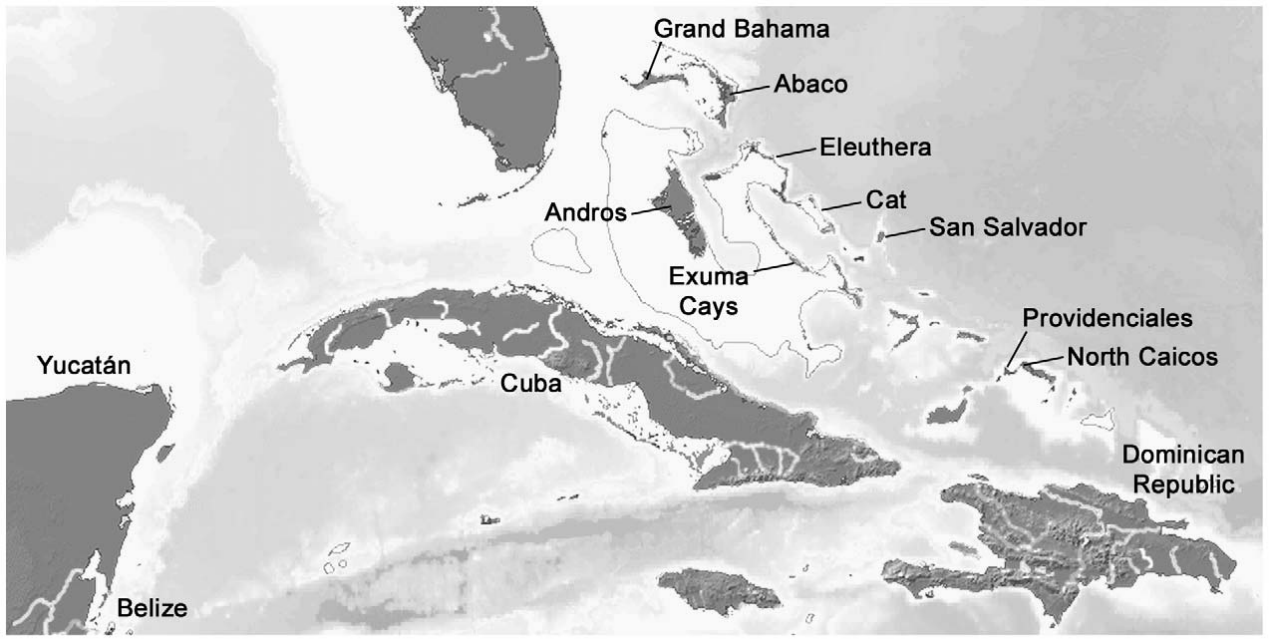
Map of the larger Caribbean region. Names of islands and regions, where Remipedia occur are indicated. For detailed information on the distribution of Remipedia see Figure 4. Map (modified) with kind permission of Demis (www.demis.nl).

The known nectiopodan remipedes exhibit a disjunct global distribution pattern (Figure 6), with the highest abundance and diversity in the Caribbean region, and isolated species in the Canary Islands and in Western Australia. Within the greater Caribbean region (Figure 7), the Bahamas Archipelago, including the Bahamas and the Turks and Caicos Islands, stands out as the center of biodiversity. This region has two endemic families (Figure 4). Godzilliidae consists of five described species: *Godzillius robustus* Schram et al., 1986 [14], *Godzilliognomus frondosus* Yager, 1989 [45], *Godzilliognomus schrami* Iliffe et al., 2010 [51], *Pleomothra apletocheles* Yager, 1989 [46] and *Pleomothra fragilis* Koenemann et al., 2008 [52]. The monotypic Micropacteridae, with *Micropacter yagerae* Koenemann et al., 2007 [45], is exclusively known from the Turks and Caicos Islands. Of the four currently accepted genera in the family Speleonectidae, two are also known from the Bahamas Archipelago, the genus *Cryptocorynetes* Yager, 1987 [53] from the Bahamas Islands, including the three described species *Cryptocorynetes haptodiscus* Yager, 1987 [53], *Cryptocorynetes longulus* Wollermann et al., 2007 [54] and *Cryptocorynetes elmorei* Hazerli et al., 2009 [55], and the monotypic genus *Kaloketos* Koenemann et al., 2004 [56] from the Turks and Caicos Islands.

The remaining speleonectid genera, *Speleonectes* and *Lasionectes*, have wider distribution ranges. *Speleonectes* has an amphi-Atlantic distribution. *Speleonectes ondinae* (García-Valdecasas, 1984) [57] and *Speleonectes atlantida* Koenemann et al., 2009 [58] are endemic to the Corona lava tube on the Canarian Island of Lanzarote. One species each is known from the Yucatán Peninsula (*Speleonectes tulumensis* Yager, 1987 [59]), Cuba (*Speleonectes gironensis* Yager, 1994 [60]), and the Dominican Republic (*Speleonectes emersoni* Lorentzen et al., 2007 [61]). An additional seven species have been described from the Bahamas Archipelago (*Speleonectes lucayensis* Yager, 1981 [1], *Speleonectes benjamini* Yager, 1987 [53], *Speleonectes epilimnius*, *Speleonectes minnsi* Koenemann et al., 2003 [62], *Speleonectes parabenjamini* Koenemann et al., 2003 [62], *Speleonectes tanumekes* Koenemann et al., 2003 [62] and *Speleonectes kakuki*). The genus *Lasionectes* shows an even greater distribution gap, with *Lasionectes entrichoma* Yager and Schram, 1986 [15] known from several anchialine caves on the Turks and Caicos Islands, and *Lasionectes exleyi* from Bundera Sinkhole, an anchialine cave on the Western Australian Cape Range Peninsula.

### Hypotheses concerning disjunct global distributions of anchialine faunas

Disjunct global distribution patterns similar to those described for Remipedia are also observed in other anchialine stygiobionts, including atyid shrimps, thermosbaenaceans, hadziid amphipods, thaumatocypridid ostracodes, cirolanid isopods, calanoid copepods in the families Epacteriscidae Fosshagen, 1973 [63], Pseudocyclopiidae Scott, 1894 [64] and Ridgewayiidae Wilson, 1958 [65] as well as members of the copepod (sub-) families Halicyclopinae Kiefer, 1927 [66], Speleophriidae Boxshall and Jaume, 2000 [67] and Superornatiremidae Huys, 1996 [68], see, e.g., [58], [69]–[71]. According to Humphreys and Danielopol [69], members of the above-mentioned taxa constitute a characteristic fauna of epicontinental anchialine cave systems, which they termed “remipede communities”. Anchialine waters on isolated seamount islands have a different faunal composition, a “procaridid community” [69], which includes species from the decapod families Alpheidae Rafinesque, 1815 [72], Hippolytidae Bate, 1888 [73], Atyidae De Haan, 1849 [74], and most characteristically from the eponymous genus *Procaris* Chace and Manning, 1972 (Procariidae Chace and Manning, 1972) [75].

Despite these differences, the remipede and procaridid communities have several genera in common. For example, various species in the thaumatocypridid genus *Danielopolina* Kornicker and Sohn, 1976 [76] are found in remipede communities in the Bahamas, Lanzarote, and the Yucatán and Cape Range Peninsulas, while congeners also occur in procaridid communities on Christmas Island [70], [77]. Another species is known from the bathyal of the South Atlantic, although the deep-sea representative may belong to a different genus [78]. Similarly, twelve species in the atyid genus *Typhlatya* are known from remipede communities in the Caribbean and one species each from procaridid communites on Bermuda, Ascension and the Galapagos Islands. Additionally, two species occur in freshwater habitats in Spain and in Herzegovina [79].

Several hypotheses have been proposed to explain disjunct global distribution patterns in hypogean crustaceans. The five main models consider vicariance, regression, deep-sea origin, active migration, and passive migration. In the vicariance model, the observed present-day disjunct distribution is regarded as a relict of a global Tethyan distribution in the Mesozoic era [67], [78], [80]–[87]. Under this scenario, range fragmentation by plate tectonics (vicariance) was followed by allopatric speciation from ancestral populations that had been widely distributed along Mesozoic shores. The regression model [88], [89] suggests that the ancestors of modern stygobionts were isolated as a result of tectonic uplift and/or eustatic lowerings of sea level followed by subsequent adaptation to brackish or limnic groundwater habitats [90]. The deep-sea hypothesis considers the possibility that caves and deep-sea environments may be linked by crevices and fissures [91]–[93], such that modern members of anchialine cave communities could be descendants of deep-sea organisms pre-adapted to total darkness and habitat with low food availability and stable environmental conditions, e.g., low temperature fluctuations. The active migration model [94], [95] proposes that some groups of anchialine organisms stem from shallow-water forms that actively colonized empty niches, such as anchialine caves and deep-sea environments, within their geographic ranges, independent of geological and climatic variations [70], [90]. The possibility of passive dispersal across oceans by currents has regained currency, fueled by findings of anchialine faunas on isolated oceanic islands [77].

Remipedes are generally assumed to be of ancient origin [14], [23], [44], [68], and their distribution range lies within the Tethyan realm [67]; however, it does not follow a “full Tethyan track” [82] because no Remipedia are yet known from the Mediterranean basin or the eastern Indian Ocean (Figure 6). In contrast to the eastern Indian Ocean, anchialine caves in the Mediterranean are well-explored, and, if a Tethyan relict distribution is assumed, the absence of Remipedia there is somewhat surprising. Although evidence is lacking, Remipedia might once have occurred in the Mediterranean basin but have become extinct, for example, in the course of the drastic geological and climatic changes associated with the Messinian salinity crises during the Miocene (reviewed in [96]).

Under the vicariance hypothesis, we would expect molecular phylogenetic reconstructions to divide the speleonectids into a Caribbean, a Canarian, and an Australian clade. Our Bayesian analysis of CO1 sequences (Figure 2) does not unambiguously support vicariance. Although the Western Australian *Lasionectes exleyi* is consistently resolved as sister taxon to all remaining speleonectids plus *Pleomothra*, the Canarian taxa are nested deeply within a clade containing only Caribbean species. This suggests that either a) several Caribbean and the Canarian lineages split before the opening of the Atlantic Ocean, or b) the amphi-Atlantic distribution of speleonectid remipedes resulted from long distance dispersal by ocean currents. Our preliminary results also suggest that dispersal may have played a major role within the Caribbean region, however, the influence of local dispersal and sea-level changes in this region still awaits investigation.

### Assessing the biodiversity of Remipedia

We are describing newly discovered remipedes at the rate of 1 to 2 species per year, and since 2002, the number of described species has doubled. Given this rate of discovery, the known taxa may represent just the “tip of the iceberg” of remipede diversity and as yet unknown remipedes may be discovered in unexplored cave systems in Cuba, Jamaica, and on other West Indian islands. In addition, we have detected cryptic species based on DNA sequence data in well-explored caves, including the Canarian Island of Lanzarote (*Speleonectes atlantida* [58]), in the Bahamas (*Godzilliognomus* Yager, 1989 [46], *Speleonectes*, and *Godzillius* Schram et al., 1986 [14]; Figure 2) and on the Yucatán Peninsula (*Speleonectes*; Figure 2). In each case, these species are highly similar in morphology to previously described species. The detection of co-occurring cryptic species suggests that sympatry is the rule rather than the exception for this group. At present, sympatric species of Remipedia are known from nine localities, many of which host four to six recorded taxa (Figure 4). Furthermore, the presence of *Speleonectes kakuki* in a fully marine sub-seafloor cave [12] and *Speleonectes epilimnius* in the surface water of an anchialine cave in the Bahamas [16] indicates that additional species may remain to be discovered outside of the typical anchialine cave environment.

Our research collection contains a number of single, damaged and/or immature specimens that most likely represent eight as yet undescribed species, and up to four cryptic species. Based on our data, we estimate that the number of undiscovered remipede species lies between 20 and 50. However, the true number of species may be considerably higher if remipedes are present in the largely unexplored eastern Indian Ocean.

## Material and methods: molecular-phylogenetic analysis

### Choice of taxa

For a preliminary molecular phylogenetic analysis based on cytochrome oxidase c subunit 1 (CO1) sequence data, we used specimens of 17 described and five as yet undescribed species of Remipedia, representing two families and six genera. In addition, we selected four outgroup taxa as representatives of higher crustacean and hexapod lineages to evaluate their possible sister-group relationships to Remipedia, including *Penaeus monodon* Fabricius, 1798 [97] (Malacostraca), *Branchinella occidentalis* Dakin, 1914 [98] (Branchiopoda), *Hutchinsoniella macracantha* Sanders, 1955 [11] (Cephalocarida) and *Camopdea tillyardi* Silvestri, 1931 [99] (Hexapoda) (see Table 1).

**Table 1:** List of taxa used for phylogenetic analysis, including GenBank accession numbers (Acc. no.) of CO1 sequences and voucher numbers of specimens in the study collection of S. Koenemann; newly generated sequences are shown in bold type.

	Species	Acc. no. CO1	Voucher	Collection Site
**Remipedia**				
Godzilliidae	*Godzillius robustus*	**JF332152**	03-19	Cottage Pond
	*Godzillius* n. sp. A	**JF332153**	AB-06-RS1	Ralph's Sink
	*Godzilliognomus frondosus*	FJ527839	06-048-4	Dan's Cave
	*Godzilliognomus schrami*	**JF332154**	07-048-2	Windermere Abyss
	*Pleomotra apletocheles*	FJ527840	AB06-DC-5.1	Dan's Cave
	*Pleomothra* n. sp. E	**JF332155**	07-038	Figure Eight Blue Hole
Speleonectidae	*Cryptocorynetes elmorei*	**JF332156**	07-035B	Bung Hole
	*Cryptocorynetes haptodiscus*	FJ527837	AB06-SS-1.1	Sawmill Sink
	*Cryptocorynetes longulus*	**JF332157**	C3-04-23	Big Fountain Blue Hole
	*Lasionectes entrichoma*	**JF332158**	03-16	Cottage Pond
	*Lasionectes exleyi*	**JF332159**	BES-10169	Bundera Sinkhole
	*Speleonectes atlantida*	FJ905040	LZ 2.3	Corona lava tube
	*Speleonectes benjamini*	FJ527841	06-047-2	Dan's Cave
	*Speleonectes emersoni*	**JF332161**	05-020-01	Cueva Taína
	*Speleonectes gironensis*	AF370851	–	Cueva de los Carboneros^1^
	*Speleonectes kakuki*	**JF332163**	04-021-1	Gaitor's Blue Hole
	*Speleonectes lucayensis*	**JF332160**	AB06-LR-1	Lost Reel Blue Hole
	*Speleonectes* n. sp. A	**JF332164**	AB-06-047-6	Dan's Cave
	*Speleonectes* n. sp. W	**JF332162**	08-033-4	Sawmill Sink
	*Speleonectes* n. sp. X	JF297644	09-005	Cenote Crustacea
	*Speleonectes ondinae*	FJ905037	LZ 1.2	Corona lava tube
	*Speleonectes tulumensis*	AY456190	–	unknown
**Outgroup**				
Branchiopoda	*Branchinella occidentalis*	EF189664	–	–
Cephalocarida	*Hutchinsoniella macracantha*	AY456189	–	–
Malacostraca	*Penaeus monodon*	AF217843	–	–
Hexapoda	*Campodea tillyardi*	AF370844	–	–

See Figure 4 for information on collection sites and geographic distribution ranges.

1 pers. comm. G. Giribet.

### Newly generated sequence data

Total genomic DNA was extracted from leg or trunk tissue of each remipede according to the manufacturer's protocol of the QIAGEN DNeasy Blood & Tissue Kit. Polymerase chain reaction (PCR) was used to amplify fragments of the CO1 gene. Our PCR forward primer, T7MH51, included LCOI-1490 [100], and a universal T7 primer (5′-TAA TAC GAC TCA CTA TAG GGT AAA CTT CAG GGT GAC CAA AAA ATC A-3′); the reverse PCR primer, SP6MH50, was a combination of HCOI-2198 [100] and Sp6 (5′-ATT TAG GTG ACA CTA TAG AAT GGT CAA CAA ATC ATA AAG ATA TTG-3′). The PCR products were purified using the NucleoSpin Extract II Kit from Macherey-Nagel, and bidirectionally sequenced by Macrogen (Korea) using the primers Sp6 (5′-ATT TAG GTG ACA CTA TAG AAT-3′) and T7 (5′-TAA TAC GAC TCA CTA TAG GG-3′). The annealing temperature for PCR and sequencing reactions was 50°C; size and quality of both PCR and purified products were examined on a 1.4% agarose gel, stained with ethidium bromide. Sequences were assembled with Seqman II (DNASAR Lasergene software) and aligned with MUSCLE [101]. Sequences were deposited in GenBank (see Table 1).

### Phylogenetic analysis

We used MrBayes 3.1.2 [102] to analyze the CO1 data set (657 bp ranging from position 46 to 702 in complete CO1 sequence of *Speleonectes tulumensis*; GenBank accession number AY456190; [30]). We applied a codon model (invertebrate mitochondrial genetic code) implemented in MrBayes 3.1.2 based on the formulations outlined in [103], [104]. Nucleotide changes were modelled using a general time reversible model assuming a Γ–shaped rate variation across sites and a proportion of invariable sites (GTR+Γ+I; [105], [106]) according to the results of the Akaike Information criterion (AIC; [107]) in MrModeltest v2.3 [108]. Bayesian analysis (BA) was performed in MrBayes 3.1.2 using a single run with four chains (one cold and three heated) for 30,000,000 generations. Trees were sampled every 1000^th^ generation. Stationarity was reached after 20,000,000 generations. Therefore, the 50% majority-rule consensus tree (Figure 2) was summarized using the last 10,001 sampled trees. Tracer v1.4.1 [109] was used to determine the burn-in proportion and to check convergence of parameter estimates. The effective sample size (ESS) value of each estimated parameter exceeded the recommended threshold of 200.
